# Living with facioscapulohumeral muscular dystrophy during the first two COVID-19 outbreaks: a repeated patient survey in the Netherlands

**DOI:** 10.1007/s13760-023-02443-3

**Published:** 2024-01-13

**Authors:** Johanna C. W. Deenen, Joost Kools, Anna Greco, Renée Thewissen, Wiecke van de Put, Anke Lanser, Leo A. B. Joosten, Andre L. M. Verbeek, Baziel G. M. van Engelen, Nicol C. Voermans

**Affiliations:** 1 Department of Neurology, Donders Institute for Brain, Cognition and Behaviour, Radboud University Medical Center, P. O. Box 9101, 6500 HB Nijmegen, The Netherlands; 2 Department for Health Evidence, Radboud University Medical Center, P. O. Box 9101, 6500 HB Nijmegen, The Netherlands; 3 Patient Representative and Chairman FSHD Advocacy Group, Patient Organization for Muscular Disease Spierziekten Nederland, Lt. Gen. van Heutszlaan 6, 3743 JN Baarn, The Netherlands; 4 Department of Internal Medicine, Radboud University Medical Center, P. O. Box 9101, 6500 HB Nijmegen, The Netherlands; 5 Department of Medical Genetics, Iuliu Hatieganu University of Medicine and Pharmacy, Strada Victor Babeș 8, 400347 Cluj-Napoca, Romania

**Keywords:** Neuromuscular diseases, COVID-19, Epidemiology, Surveys and questionnaires, Incidence, Registries

## Abstract

**Background:**

Patients with facioscapulohumeral dystrophy (FSHD) suffer from slowly progressive muscle weakness. Approximately 20% of FSHD patients end up wheelchair-dependent. FSHD patients benefit from physical activity to maintain their muscle strength as much as possible. The impact of the COVID-19 pandemic on the health of FSHD patients was unknown.

**Objective:**

This study assessed changes in daily care received, perceived psychosocial stress, and worsening of FSHD complaints in 2020. Furthermore, we compared COVID-19 infection incidence and severity of symptoms between FSHD patients and non-FSHD housemates.

**Methods:**

Three online survey rounds were sent out to all adult participants of the Dutch FSHD registry regarding daily care received, perceived psychosocial stress, COVID-19 infection rate, and COVID-19 symptoms severity. They also included COVID-19-related questions regarding the participants’ housemates, which served as control group.

**Results:**

Participation rate was 210 (61%), 186 (54%), and 205 (59%) for survey 1, 2, and 3, respectively. Care reduction was reported by 42.7%, 40%, and 28.8% of the participants in the respective surveys. Perceived psychosocial stress increased in 44%, 30%, and 40% of the participants. Compared to the 197 non-FSHD housemates, the 213 FSHD patients reported more possibly COVID-19-related symptoms (27% vs. 39%, *p = *0.017) of mostly minimal severity (63%). No difference in (possible) COVID-19 infection incidence rates was found (2.0% vs. 2.8%, *p = *0.527).

**Conclusions:**

The COVID-19 pandemic negatively impacted care received and increased perceived psychosocial stress in FSHD patients. However, COVID-19 infection incidence in FSHD patients was similar to their non-FSHD housemates.

**Supplementary Information:**

The online version contains supplementary material available at 10.1007/s13760-023-02443-3.

## Introduction

The coronavirus disease 2019 (COVID-19) pandemic has affected the health status, daily activities, social participation, care availability, and quality of life of individuals all over the world. In the Netherlands, 6.5 million people tested positive in a registered PCR test, and almost 40,000 people died over the course of 2 years [1, 2]. To slow down the rapid spread of the disease, rigorous restrictions were implemented in March 2020 for a prolonged period of time, such as social distancing, quarantine, and lockdowns [3].

These restrictions resulted in a decrease of physical activity, available healthcare, and an increase in loneliness, anxiety, and depression [4, 5]. For patients with facioscapulohumeral muscular dystrophy (FSHD), a slowly progressive muscle disease, physical activity is crucial to maintain muscular strength, flexibility in joints, and physical endurance to reduce progression of muscle weakness [6, 7]. At the time, the study was initiated in March 2020, it was unknown what the impact of COVID-19, and the restrictions on FSHD patients would be. In Italy, research on various Neuromuscular Disorders (NMDs) has shown a subjective worsening of the NMD symptoms and a significant worsening of quality of life (QoL) during the pandemic [8, 9]. It is expected that the worsening of disease aspects and QoL will also have occurred in FSHD patients. However, the infection rate and course might differ in FSHD patients. Previous studies hypothesized that the inflammation observed in biopsies and imaging modalities could point to possible alterations in the immune responses [10, 11]. On the other hand, a minority of patients does experience respiratory weakness or weakness in coughing, increasing the susceptibility for infections [12, 13]. It is unknown whether these changes affect the response to the SARS-CoV-2 virus.

The goal of this study was twofold. First, we aimed to assess and describe the physical and mental health of the FSHD patients during the pandemic. Second, we aimed to gain more insight in the COVID-19 incidence rate and severity of symptoms compared to a non-FSHD population.

## Materials and methods

### Study design

This was an observational questionnaire study, performed in an already existing cohort (i.e., the Dutch FSHD registry cohort). A survey was created to inquire about the impact of the COVID-19 pandemic on care received, perceived psychosocial stress, FSHD complaints, the number of COVID-19 infections, and the severity of corresponding symptoms (Appendix 1 in supplementary material). The survey was electronically sent using CastorEDC to FSHD patients in three rounds in 2020: survey 1 (S1) on May 22nd 2020, survey 2 (S2) on August 26th 2020, and survey 3 (S3) on December 19th 2020 [14].

### Study population: the Dutch FSHD registry

The Dutch FSHD registry was set up in 2015 to enable recruitment of FSHD patients for research and to collect patient-reported data about the natural course of the disease, including the core dataset decided upon during the 225th European Neuromuscular Centre (ENMC) workshop [15–17]. The registry was originally intended for Dutch-speaking participants only. Other interested people were encouraged to participate in the national registry in their country. Since 2020, people who still wished to enter the Dutch registry despite geographical and language barriers were accepted in the Dutch registry.

All registered FSHD patients aged 16 years and older, the age of consent in the Netherlands regarding medical decisions, were invited for the surveys. The control group consisted of the housemates of the participants who were ≥ 16 years old and did not have FSHD. This enabled comparison of COVID-19 infection incidence rate and severity of possible COVID-19-related symptoms. Housemates were defined as: spouses, children, parents, family, or other. Housemates with FSHD were excluded from the analysis to prevent any accidental duplications in FSHD patients. The data concerning the housemates were reported by the FSHD patients instead of the housemates themselves, because no contact details of housemates were available in the registry. Furthermore, it was a relatively quick process to submit an amendment on the already existing approval of the FSHD registry. Sending the surveys directly to housemates or other control groups would have required a completely new submission, which would have delayed the study. As time was of the essence during the pandemic, the method for gathering indirect data on housemates was chosen.

### Survey

Demographic data regarding age and sex were retrieved from the Dutch FSHD registry. Furthermore, the survey contained a question about risk factors for a more severe COVID-19 disease course known at that time: age > 70 years, respiratory problems, chronic heart disease, severely overweight, and immunodeficiency.

The survey consisted of three parts: (1) impact of the pandemic on FSHD complaints and care (2) perceived psychosocial stress, and (3) COVID-19 infection rate and severity of possible symptoms experienced by the FSHD patients and their housemates.

Specifically, part one consisted of questions concerning the participants’ living arrangement, care received pre-COVID-19, change in received care during the pandemic compared to pre-pandemic care received (yes/no answer with option to elaborate on what changed and the consequences of the changes), and the Modified Ranking Scale (MRS) [18]. The MRS asks about the disease severity as experienced by the participants with 0—‘no symptoms’ and 5—‘severely handicapped, constant need for care’. Participants were asked to report the MRS pre-pandemic and at the time of survey completion.

The second part consisted of questions about the perceived psychosocial stress during the pandemic compared to before (0 ‘a lot less stress’—5 ‘a lot more stress’). It included the Perceived Stress Scale (PSS) ranging from 0 ‘no stress’ to 40 ‘severe stress’, which evaluates how unpredictable, uncontrollable, and overloading someone experienced the previous month, and their perceived ability to cope [19]. Furthermore, a set of possible COVID-related stressors used in an ongoing global study were tested on percentage (I do/do not experience this stressor) and their associated burden if experienced (0 ‘no burden’—5 ‘high burden’). Finally, participants were asked to report on any positive effects of the pandemic (yes/no answer with option to elaborate on what positive effect if present) [19–21].

Part three inquired whether participants and housemates experienced COVID-19-related symptoms suggestive of an infection and the severity of these symptoms, as well as if they were tested for COVID-19 and the result of the test.

### COVID-19 timeline and survey modifications

**Fig. 1:**
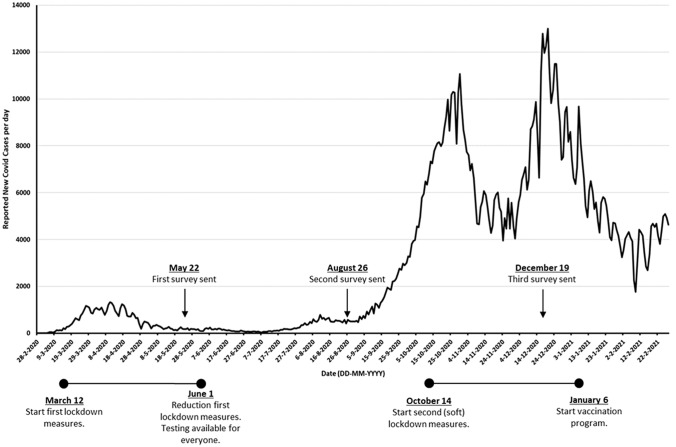
New COVID-19 infections per day in the Netherlands during the pandemic. The timepoints when the surveys were sent are pictured in the graph. The most important restrictions and developments regarding testing are stated below the graph [22]. Dates are given as dd-mm-yyyy

Each country reacted differently to the COVID-19 pandemic with restrictions and opportunities changing over time. A timeline with the number of COVID-19 infections and the most important events in the Netherlands in 2020 is shown in Fig. 1. During the first months of the pandemic, testing facilities were only available in case hospitalization was needed and primary healthcare availability was limited due to lockdown restrictions. This period coincided with survey 1. From June 2020 on, access to both testing facilities and primary healthcare became available again across the country. Furthermore, barely any restrictions regarding the pandemic were present when survey 2 was sent. At the time of the last survey, new restrictions in the form of a soft lockdown were present and (self)testing on COVID-19 was widespread available. Because of these changes, slight modifications to questions concerning COVID-19 incidence and testing were made in survey 2 and 3 to fit the new situation, mostly concerning questions regarding testing of COVID-19 (Appendix 2 in supplementary material).

During survey 1, a large portion of the participants reported reduced physical activity in the comment sections of questions. Therefore, a question was added to capture this in survey 2 and 3.

### Data availability and analysis

The data supporting the findings of this study are available on request from the Dutch FSHD registry. The data are not publicly available due to privacy or ethical restrictions [15]. Data were collected in CastorEDC [14]. Analysis of the data was done in R (R Foundation for Statistical Computing, Vienna, Austria) and SPSS (IBM Corp. Released 2017. IBM SPSS Statistics for Windows, Version 25.0. Armonk, NY: IBM Corp.). Figures were created using GraphPad Prism version 9.0.0 for Windows (GraphPad Software, San Diego, California USA).

Demographics, impact of the pandemic on care, and perceived psychosocial stress are reported using descriptive statistics. The received care pre-pandemic is reported as a pooled group of all unique patients across the three surveys. Data are reported as mean (SD) or median [IQR] depending on normality of the data. Pearson’s chi-square was used to test for differences between FSHD patients and the non-FSHD housemates concerning COVID-19 infection rate and severity of the symptoms with a *p* value < 0.05 considered as statistically significant. These analyses were done using only data of survey 3, because for this survey, patients had to report on the whole period since the start of the pandemic, including the timespans of survey 1 and 2. Furthermore, for this comparison, only housemates ≥ 16 years were included.

### Ethical approval and informed consent

This study involved clinical research that did not fall within the scope of the Medical Research Involving Human Subjects Act, as declared by the local Medical Ethics Review Committee of the Radboud university medical center (amendment of file 2015-1812 on April 15th 2020). All participants of the FSHD registry provided their written informed consent before they entered the registry. The registry and its databases are in concordance with the General Data Protection Regulation and all other acting laws.

## Results

### Demographics and clinical features

Of the respectively 339, 341 and 343 invited patients for each for the three surveys, 210 (62%) completed the first, 186 (55%) the second, and 205 (60%) the third survey. In total, 261 participants completed at least one survey. The mean age per survey ranged from 54.6 (14.1) to 56.0 (14.5) years and 39–44% of the population was male (Table 1). Almost half of the participants in each survey (47.6% (S1), 49.5% (S2), and 46.8% (S3)) belonged to one or several risk groups for a severe course of COVID-19 when infected with the SARS-CoV-2 virus.

**Table 1:** Demographics by survey round

	Survey 1 (22 May 2020)	Survey 2 (26 Aug 2020)	Survey 3 (19 Dec 2020)	Non-FSHD housemates
*N*	210	186	205	204
Age, mean (SD)	54.6 (14.1)	56.0 (14.1)	55.7 (14.5)	49.9 (18.3)^b^
Male	82 (39)	78 (42)	90 (44)	106 (52)
Living arrangement^a^				
Independent	169 (81)	155 (83)	170 (83)	
Home care or personal care budget	25 (12)	19 (10)	24 (12)	
Assisted living or care facility	7 (3)	5 (3)	5 (2)	
Other	9 (4)	7 (4)	6 (3)	
Risk factors severe COVID-19				
> 70 years old	33 (16)	36 (19)	36 (18)	22 (11)
Respiratory problems	27 (13)	24 (13)	21 (10)	5 (2)
Chronic heart disease	18 (9)	15 (8)	12 (6)	7 (3)
Severely overweight^b^	7 (3)	5 (3)	6 (3)	6 (3)
Immunodeficient	7 (4)	7 (4)	6 (3)	1 (1)
Other	36 (17)	32 (17)	34 (17)	13 (6)
Relation				
Spouse				153 (75)
Parent				10 (5)
Child				14 (7)
Brother/Sister				2 (1)
Other				7 (3)
Missing				17 (8)

Data are shown as *N* (%) unless given otherwise

^a^Independent—living independently in their own home, by themselves or with their partner/family. Home care—care at home provided by an organization, consisting of healthcare, nursing, domestic help, and guidance in everyday life; Personal care budget—a budget provided by the government with which a patient can buy their own care or assistance; Assisted living or care facility: a house or institution in which the patient lives and is provided with daily care, such as a nursing home

^b^Housemates were significantly younger compared to FSHD patients of survey 3 (*p < *0.001)

### COVID-19 impact on received care, FSHD complaints, and physical activity

**Fig. 2:**
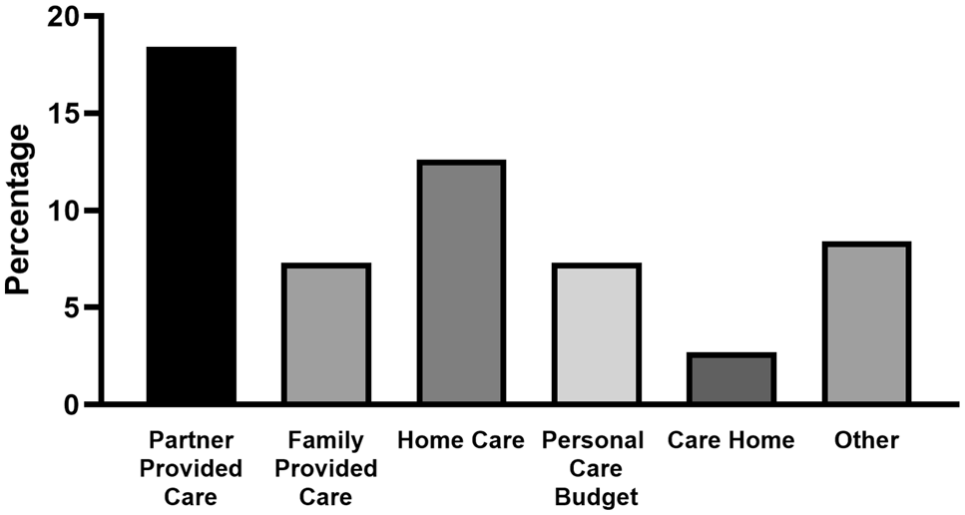
Types of care participants (*N = *86) reported to receive under normal circumstances. Of the 261 unique responders across the three surveys, 86 (33%) reported that they received care before the COVID-19 pandemic. Partner provided care: partner of patient provides daily care; family provided care: family provides daily care; home care: care at home provided by an organization, consisting of healthcare, nursing, domestic help and guidance in everyday life; Personal care budget: a budget provided by the government with which a patient can buy their own care or assistance; care home: a house or institution in which the patient lives and is provided with daily care, such as a nursing home

Pre-pandemic care was received by 86 (33%) participants across the three surveys, mostly consisting of care from their partner (18.4%) and/or homecare (12.6%) (Fig. 2). At the time of surveys 1 and 2, 41.7% and 40% of the patients receiving care reported a decrease in care received compared to pre-pandemic care, reducing to 28.8% at the time of survey 3. The following changes were most often reported: home care unavailable, physical therapy unavailable, care personnel having less time, and domestic help unavailable. This reportedly led to a higher burden for informal caregivers, more symptoms, and less activity in general. Although an increase in FSHD-related symptoms was reported by participants, the pre-pandemic MRS did not differ from the MRS at time of the survey [*p = *0.99 (S1), *p = *0.99 (S2), and *p = *0.90 (S3)]. In surveys 2 and 3, 45% and 53% of the participants, respectively, were a little to a lot less active compared to before the pandemic.

### Impact of the pandemic on perceived psychosocial stress

**Fig. 3:**
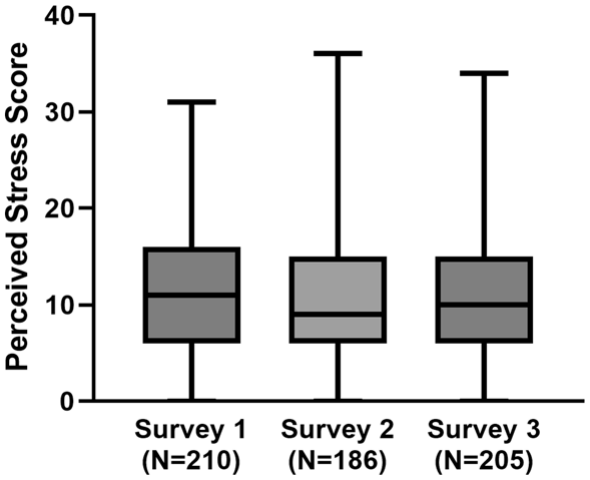
Perceived Stress Scale of participants from three consecutive survey rounds. A total score of 0–13 is considered low stress, 14–26 moderate stress, and 27–40 high stress

Compared to pre-pandemic perceived psychosocial stress (PSS), 44% (S1), 30% (S2), and 40% (S3) of the participants reported a little to a lot more stress. Nevertheless, the perceived stress scores were low, with a median PSS of 11 [6–16] (S1), 9 [6–15] (S2), and 10 [6–15] (S3) (Fig. 3). Stressors most often reported were ‘loss of social contact’ (86–91.4%) and ‘COVID-19 related media coverage’ (89.3–90.3%). The stressors that were most burdensome for FSHD patients were ‘being unable to attend a funeral of a loved one’ (3.06 (1.25) – 3.57 (1.16)) and ‘being restricted in visiting family, friends or loved ones in the hospital’ (3.03 (1.00) – 3.23 (1.16)) (Appendix 3 in supplementary material).

Positive effects of the pandemic were reported by 32.4% (S1), 26.3% (S2), and 27.8% (S3) of the participants. The most often reported positive effects were fewer social obligations and more time to rest resulting in less pain, less fatigue, less stress, and the opportunity to spend more time with their partners and children.

### Comparison FSHD patients and their housemates

In survey 3, 216 housemates were reported on of which 12 housemates were also FSHD patients, resulting in 204 non-FSHD housemates (Table 1). The housemates were significantly younger compared to the FSHD patients [49.9 (18.3) vs. 55.7 (14.5) years old, *p < *0.001]. The majority of the housemates were the spouse of the FSHD patients (*n = *153, 75%), followed by their children (*n = *14, 7%) and parents (*n = *10, 5%).

**Fig. 4:**
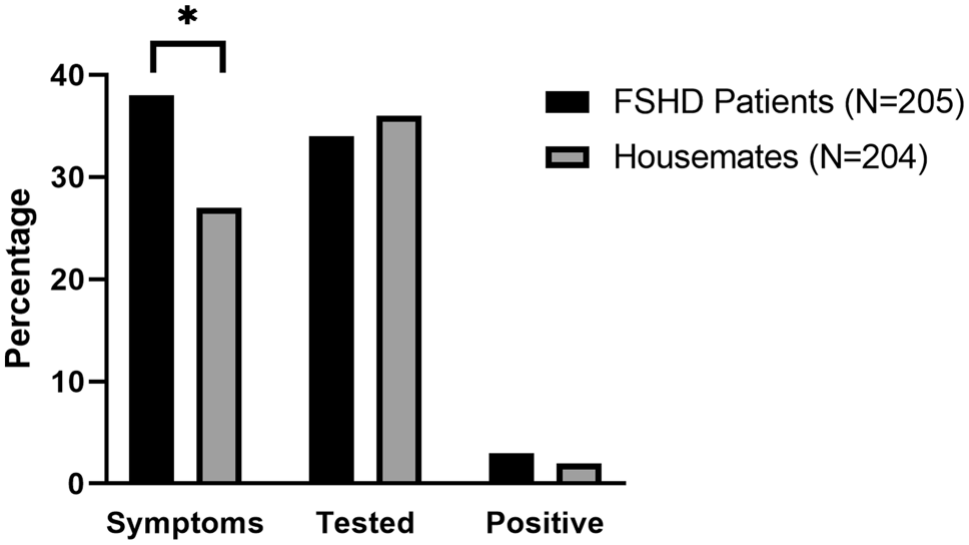
Comparison of possible COVID-19 symptoms, COVID-19 tests performed, and number of positive tests in FSHD patients versus their housemates. Results are based only on survey 3. FSHD patients reported significantly more possible COVID-19-related symptoms (38.5% vs 27.4%, *χ*^2^ = 5.68, *p = *0.017). There was no difference between the number of tested participants (regardless of positive or negative result) (33.3% vs 35.5%, *χ*^2^ = 0.219, *p = *0.639) and number of positive tests (2.8% vs. 2.0%, *χ*^2^ = 0.40 *p = *0.527)

**Fig. 5:**
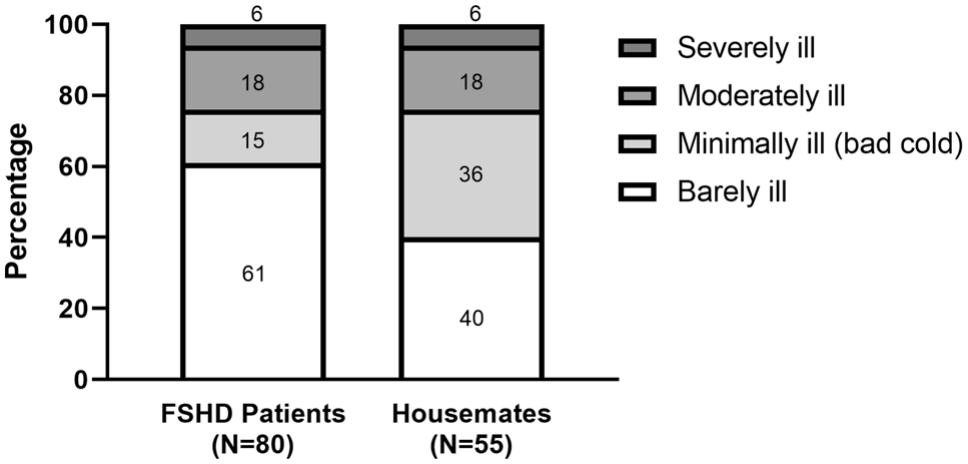
Severity of possible COVID-19-related symptoms in FSHD patients compared to their housemates. The percentages were calculated based on the number of FSHD patients and housemates who experienced symptoms (*N = *80 and *N = *55, respectively). The severity differed significantly between the two groups (*N = *136, *χ*^2^ = 10.34, *p = *0.016)

FSHD patients had more possible COVID-19-related symptoms (38% (*n = *80) vs 27% (*n = *55), *χ*^2^ = 6.73, *p = *0.012). No differences were found in the number of patients and housemates that were tested [34% (*n = *70) vs 36% (*n = *74), *χ*^2^ = 0.203, *p = *0.68] or tested positive [3% (*n = *6) vs. 2% (*n = *4), *χ*^2^ = 0.558 *p = *0.53] (Fig. 4). The severity of possible COVID-19-related symptoms differed significantly between patients and their housemates (*N = *135, *χ*^2^ = 9.11, *p = *0.03) (Fig. 5).

## Discussion

This study investigated the impact the COVID-19 pandemic had on FSHD patients and the incidence of COVID-19 infections in the Netherlands. The COVID-19 pandemic reduced available care, physical activity, and increased the psychosocial stress in FSHD patients. The COVID-19 infection rate in FSHD patients did not differ from their housemates without FSHD, but they did report more symptoms of minimal severity.

At surveys 2 and 3, nearly 50% of the patients reported to be less active during the pandemic than before. This is a considerable difference with findings in the general population, where no decline of physical activity was observed [23]. We hypothesize that people without physical challenges can easily change to outdoor activities, which may be harder to do for patients with FSHD or other NMDs. Since physical activity is known to be an important factor to stay in shape for FSHD patients, it is important to educate and support patients in maintaining their levels of physical activity during another pandemic. Even though face-to-face interactions are preferred by patients, during a pandemic, this might not be possible and telemedicine approaches should be considered for the continuity of physical therapy and rehabilitative care [24–26].

Patients reported to have more psychosocial stress than before the pandemic. This was not reflected by the PSS scores reported in our study, which were low compared to worldwide studies in the general population as well in NMD patients during the pandemic (PSS scores of 15.4 to 17.4) [13, 27, 28]. However, similarly low PSS scores were also reported from the general population in the Netherlands in the same time during the pandemic [23]. The lower stress scores might be due to a higher social security and relatively mild course of the pandemic in the Netherlands compared to other countries. Studies with longer follow-up periods will need to confirm if the stress levels of patients normalize to pre-pandemic levels.

The most prevalent and most burdensome stressors in our study were similar to stressors in healthy individuals (DYNACore-C) and in Parkinson’s patients, indicating that the stressors perceived by FSHD patients were not disease-specific [20, 29]. Findings from large studies on these stressors such as the DYNACore-C may therefore be applicable to FSHD patients, which might help with creating therapies to cope with these stressors. Interestingly, more than 25% of the FSHD patients from each survey reported various positive effects of the pandemic, for instance being well rested. A more detailed, possibly qualitative, follow-up on what these positives effects were may help us to improve the quality of life of FSHD patients within as well as outside of a pandemic period.

We did not find a difference in infection incidence rates between FSHD patients and their non-FSHD housemates. One international study in 1243 NMD patients reported a higher infection rate of 8% compared to our findings, but only a minority of those infections (20%) were found in European patients bringing it more in line with our incidence rate [3]. Another international study mentioned an infection incidence of < 1% but lacked details [13]. Our data did show a higher incidence of possible COVID-related symptoms in FSHD patients compared to their housemates. However, we suspect that this is due to reporting bias as recalling one’s own minimal symptoms is different from identifying and recalling when housemates experienced such symptoms. We also suspect that the higher number of minimal symptoms in the FSHD patients caused the difference in severity of symptoms between the patients and their housemates.

Due to the limitations of social distancing and lockdowns as well as the lack of contact details of participants’ spouses in the registry and limitations in the survey system, the study was limited to data reported by the registry participants, including the data about the housemates. Therefore, a drawback of this method is that the data on housemates is secondhand information and might be more biased. In addition, although we did inquire about the exposure by asking participants about measures taken, we failed to ask about the situation of the housemates. Therefore, we cannot rule out possible exposure differences between participants and housemates.

This study assessed the changes in health(care) during the pandemic. The healthcare system changed after the pandemic, most noticeably in the higher frequency of telemedicine approaches. A study comparing pre- and post-pandemic healthcare received and the satisfaction regarding the new telemedicine approach would be interesting to perform.

## Conclusion

This study showed that care received, physical activity, and perceived psychosocial stress were negatively impacted by the COVID-19 pandemic. Although an increase in FSHD complaints was reported by participants, the pre-pandemic MRS did not differ from the MRS at time of the survey. We did not find evidence for a different susceptibility to COVID-19 infections in FSHD patients compared to the control group and differences in the number and severity of possible COVID-19-related symptoms could well be attributable to reporting bias. Since the COVID-19 pandemic is characterized by cyclical outbreaks and given the possibility for other future pandemics, an adequate approach for the support and continuity of care of these patients is essential.

### Supplementary Information

Below is the link to the electronic supplementary material.

Supplementary file1 (PDF 298 kb)

Supplementary file2 (PDF 150 kb)

Supplementary file3 (PDF 143 kb)

## Abbreviations

FSHD: Facioscapulohumeral muscular dystrophy
COVID-19: Coronavirus disease 2019
NMD: Neuromuscular disorder
QoL: Quality of life
ENMC: European Neuromuscular Centre
MRS: Modified Ranking Scale
PSS: Perceived Stress Scale
