# Supplementary material for: Living with facioscapulohumeral muscular dystrophy during the first two COVID-19 outbreaks: a repeated patient survey in the Netherlands
Source: Acta Neurol Belg. 2024 Jan 13;124(2):559–66. doi: 10.1007/s13760-023-02443-3 (PMC10965598; doi:10.1007/s13760-023-02443-3)
Supplement: Supplementary file 1 — Supplementary file1 (PDF 298 kb) [file 13760_2023_2443_MOESM1_ESM.pdf]

Appendix 1.

Article Title: Living with facioscapulohumeral muscular dystrophy during the first two COVID-19 outbreaks, a repeated patient survey in the Netherlands

Journal Name: Acta Neurologica Belgica

Author names: Johanna C.W. Deenen[1,a,b], Joost Kools[1,a], Anna Greco[2,a,d], Renée Thewissen[2,a], Wiecke van de Put[a], Anke Lanser[c], Leo A.B. Joosten[d,e], Andre L.M.Verbeek[b], Baziel G. M. van Engelen[a], Nicol C. Voermans[a].

[1]Authors contributed equally; [2] Authors contributed equally;

[a]Department of Neurology, Donders Institute for Brain, Cognition and Behaviour, Radboud university medical center, PO Box 9101, 6500 HB Nijmegen, the Netherlands;

[b]Department for Health Evidence, Radboud university medical center, PO Box 9101, 6500 HB Nijmegen, the Netherlands;

[c]Patient Representative & Chairman FSHD Advocacy Group, Patient Organization for Muscular Disease Spierziekten Nederland, Lt. Gen. van Heutszlaan 6, 3743 JN Baarn, the Netherlands;

[d]Department of Internal Medicine, Radboud university medical center, PO Box 9101, 6500 HB Nijmegen, the Netherlands;

[e]Department of Medical Genetics, Iuliu Hatieganu University of Medicine and Pharmacy, Strada Victor Babeș 8, Cluj-Napoca 400347, Romania.

Corresponding author: Nicol.Voermans@radboudumc.nl

Correspondence author:

Nicol C. Voermans, Department of Neurology, Donders Institute for Brain, Cognition and Behaviour, Radboud university medical center, Radboud university medical center, PO Box 9101, 6500 HB Nijmegen, the Netherlands. Tel: 00 31 24 36 14 308, Fax: 00 31 24 36 35 135. Email: nicol.voermans@radboudumc.nl

Appendix 1 Covid-19 survey English version

Corona - your situation and occupations

Be careful, do not write down any traceable information

1.1 How is your living situation?
- o Independently
- o Independently with home care or care facilitated by a personal care budget
- o Assisted living (including Fokus)
- o In a care institution
- o Other,…

1.1.1 Describe your other living situation please

1.2 How many people are living in your household, including yourself?

1.3 Are you active in a ‘vital job’ according to the government rules?
- o No
- o I do not know
- o I do not want to answer
- o Yes, but I am not working
- o Yes and I am working

1.4 Which description suits you (multiple answers possible)?
- o (High) school pupil
- o Studying at a high education facility
- o Independent contractor without staff
- o Independent contractor with staff
- o Under employment
- o Stay-at-home-parent (fulltime or most of the time)
- o Volunteer
- o Retired
- o Unable to work
- o Unemployed
- o Other,…

1.4.1 Please describe your situation

1.5 Do you use personal care on a regular basis, under normal circumstances?
- o Yes
- o No

1.5.1 How is your personal care normally organised?
- o My partner/ residential family or housemates take care of me
- o My family and friends take care of me
- o I receive home care
- o I arrange my personal care via a personal care budget
- o The institution which I am living in arranges my personal care
- o Other, …

1.5.1.1 Please describe your other personal care

1.5.2 Does the personal care differ during the Corona crisis versus normal circumstances?
- o Yes
- o No

1.5.2.1 Describe the changes and consequences in your personal care since the Corona outbreak

Corona- Measurements

2.1 Which of the following alterations did you make due to the Corona outbreak?

- Staying at home/ working from home
- 1,5m distance
- Frequently washing hands with soap
- Coughing and sneezing in the inner side of the elbow
- Using paper towels
- Letting others do your groceries
- Being in a room with a maximum of two people
- Using personal protective equipment such as masks, gloves etc.
- Other,…

2.1.1 Which other measurement did you make?

2.2 Do you consider yourself to be part of one or more risk groups?

- 70 years old or older
- Adult with respiratory illness under the treatment of a doctor
- Adult with chronical heart disease under treatment of a cardiologist
- Adult with poorly managed diabetes or diabetes with complications
- Adult on dialysis or with a donor kidney
- Adult with diminished immune response against infections caused by medication in connection with auto-immune disease
- Adult with organ or stem cell transplantation
- Adult with diminished immune response due to blood diseases
- Adult with diminished immune response due to spleen removal or disfunction of the spleen
- Adult with severe immune disfunction, which are treated
- Adult with cancer, which is treated in the past 3 months with radiation or chemotherapy
- Adult with HIV infection
- Adult with severe obesity
- Other,…
- No, I do not subject to a risk group

2.2.1 Describe in which other risk group you are, please

2.3 Have you been in voluntary quarantine?

- Yes
- No

2.3.1 Please describe your quarantine measurements?

2.3.2 Since when have you been quarantining?

2.4 What are your personal reasons to leave your home (multiple answers are possible)?

- Going to work
- Walking my pet
- Exercise (like jogging or playing sports)
- Buying groceries for myself or family
- Going to a pharmacy, hospital or doctor

- Taking care of others
- Meeting with friends or family
- To prevent boredom
- I just go outside if I want to
- No particular reason
- Other, …

2.4.1 Please describe your other reasons to leave your house

2.3.4 Did you stop quarantining in the meantime?

- Yes
- No

2.3.3.1 When did you stop quarantining?

2.5 Do you have personal protective equipment (gloves, masks, protective glasses, apron)?

- No and I do not need them
- No, but I need them
- Yes, partly
- Yes, I have everything I need

2.5.1 Why do you need protective equipment?

- Because I receive care in my close surrounding
- Because I have a cold or I am ill
- Because I have Corona
- Because I belong to a risk group

2.5.1.1 Please describe why you need protective equipment

Corona - effects and your experience

3.1 How much stress did you experience during the Corona outbreak?

- Substantially less stress than usual
- Little less stress than usual
- As much stress as usual
- A bit more stress than usual
- Substantially more stress than usual

How threatening did you find the Corona virus….

3.2 … for yourself?

- Not threatening at all
- Not that threatening
- A little threatening
- Threatening
- Really threatening
- No opinion

3.3 … for your health?

- Not threatening at all

- Not that threatening
- A little threatening
- Threatening
- Really threatening
- No opinion

3.4 … for the health of your family?

- Not threatening at all
- Not that threatening
- A little threatening
- Threatening
- Really threatening
- No opinion

3.5 … for the health of your friends?

- Not threatening at all
- Not that threatening
- A little threatening
- Threatening
- Really threatening
- No opinion

Below, you will find situations in which people could find themselves due to the Corona pandemic. Could you declare if you currently experience or have experienced any of the situations listed down below, and how inconvenient the situation is/was?

3.6 Having Corona symptoms or symptoms that could be related to Corona.

- This situation did not occur
- Not inconvenient
- Barely inconvenient
- Somewhat inconvenient
- Decently inconvenient
- Really inconvenient

3.7 Corona symptoms or symptoms related to Corona in family members, friends, loved ones or colleagues.

- This situation did not occur
- Not inconvenient
- Barely inconvenient
- Somewhat inconvenient
- Decently inconvenient
- Really inconvenient

3.8 Being at increased risk for an infection (e.g., at work).

- This situation did not occur
- Not inconvenient
- Barely inconvenient
- Somewhat inconvenient

- Decently inconvenient
- Really inconvenient

3.9 Being at increased risk for a serious course of the disease in case of an infection (belonging to a so-called 'risk group').

- This situation did not occur
- Not inconvenient
- Barely inconvenient
- Somewhat inconvenient
- Decently inconvenient
- Really inconvenient

3.10 Family, friends, or loved ones being at increased risk for a serious course of the disease in case of an infection (they belong to a so-called 'risk group').

- This situation did not occur
- Not inconvenient
- Barely inconvenient
- Somewhat inconvenient
- Decently inconvenient
- Really inconvenient

3.11 Problems with access to healthcare, medication, or sanitation.

- This situation did not occur
- Not inconvenient
- Barely inconvenient
- Somewhat inconvenient
- Decently inconvenient
- Really inconvenient

3.12 (Feeling) restricted to leave your home.

- This situation did not occur
- Not inconvenient
- Barely inconvenient
- Somewhat inconvenient
- Decently inconvenient
- Really inconvenient

3.13 Loss of social contact and social events.

- This situation did not occur
- Not inconvenient
- Barely inconvenient
- Somewhat inconvenient
- Decently inconvenient
- Really inconvenient

3.14 Family, friends, or loved ones are at the hospital and you are restricted in visiting them.

- This situation did not occur

- Not inconvenient
- Barely inconvenient
- Somewhat inconvenient
- Decently inconvenient
- Really inconvenient

3.15 Unable to attend a funeral of a family member, friend, or loved one.

- This situation did not occur
- Not inconvenient
- Barely inconvenient
- Somewhat inconvenient
- Decently inconvenient
- Really inconvenient

3.16 Having family, friends or loved ones with a vital profession.

- This situation did not occur
- Not inconvenient
- Barely inconvenient
- Somewhat inconvenient
- Decently inconvenient
- Really inconvenient

3.17 Not being able to perform leisure activities.

- This situation did not occur
- Not inconvenient
- Barely inconvenient
- Somewhat inconvenient
- Decently inconvenient
- Really inconvenient

3.18 Difficulties combining work with childcare.

- This situation did not occur
- Not inconvenient
- Barely inconvenient
- Somewhat inconvenient
- Decently inconvenient
- Really inconvenient

3.19 Tensions at home or family conflict

- This situation did not occur
- Not inconvenient
- Barely inconvenient
- Somewhat inconvenient
- Decently inconvenient
- Really inconvenient

3.20 Work-related delays/obstacles.

- This situation did not occur
- Not inconvenient
- Barely inconvenient
- Somewhat inconvenient
- Decently inconvenient
- Really inconvenient

3.21 (Threat of) job loss or insolvency of private company.

- This situation did not occur
- Not inconvenient
- Barely inconvenient
- Somewhat inconvenient
- Decently inconvenient
- Really inconvenient

3.22 Problems obtaining basic needs.

- This situation did not occur
- Not inconvenient
- Barely inconvenient
- Somewhat inconvenient
- Decently inconvenient
- Really inconvenient

3.23 Corona-related media coverage

- This situation did not occur
- Not inconvenient
- Barely inconvenient
- Somewhat inconvenient
- Decently inconvenient
- Really inconvenient

3.24 Does the Corona outbreak have positive consequences for you?

- Yes
- No

3.24.1 What positive consequences do you experience?

3.25 How often have you been upset because of something that happened unexpectedly over the last two weeks?

- Never
- Almost never
- Sometimes
- Fairly often
- Very often

3.26 How often have you felt that you were unable to control the important things in your life over the last two weeks?

- Never

- Almost never
- Sometimes
- Fairly often
- Very often

3.27 How often have you felt nervous and stressed, over the last two weeks?

- Never
- Almost never
- Sometimes
- Fairly often
- Very often

3.28 How often have you felt confident about your ability to handle your personal problems over the last two weeks?

- Never
- Almost never
- Sometimes
- Fairly often
- Very often

3.29 How often have you felt that things were going your way over the last two weeks?

- Never
- Almost never
- Sometimes
- Fairly often
- Very often

3.30 How often have you found that you could not cope with all the things that you had to do, over the last two weeks?

- Never
- Almost never
- Sometimes
- Fairly often
- Very often

3.31 How often have you been able to control irritations in your life over the last two weeks?

- Never
- Almost never
- Sometimes
- Fairly often
- Very often

3.32 How often have you felt that you were on top of things over the last two weeks?

- Never
- Almost never
- Sometimes
- Fairly often

- Very often

3.33 How often have you been angered because of things that happened that were outside of your control l over the last two weeks?

- Never
- Almost never
- Sometimes
- Fairly often
- Very often

3.34 How often have you felt difficulties were piling up so high that you could not overcome them over the last two weeks?

- Never
- Almost never
- Sometimes
- Fairly often
- Very often

Corona- Have you been ill?

4.1 Did you experience the following symptoms, since the start of the Corona outbreak mid-February in the Netherlands?

- Dry coughing without mucus
- Coughing with mucus
- Taste loss
- Smell loss
- Flu-like feeling
- Dizziness
- Extra exhaustion (more than normal)
- Feverishness
- Fever (38℃ or higher)
- Headache
- No appetite
- Nausea
- Muscle pain
- Short of breath
- Breathing tightness
- Squeaking breathing
- Pain in the lungs / with breathing
- Sore throat
- Stuffed nose
- Vomiting
- Ear pain
- Eye infection
- Other, …
- No symptoms

4.1.1 What other symptoms did you experience?

4.1.2 When did you first get these symptoms (please estimate the date if you don't know precisely)

4.1.3 How bad were your symptoms?

- Minimal symptoms
- Few symptoms, having a severe cold but not sick
- Ill
- Severely ill

4.2 Were you in contact with someone who presumably has Corona?

- Yes
- No

4.1.4 Do you think you have/had Corona?

- Yes
- No
- Maybe

4.3.1 Where do you expect your contamination came from?

- Yes
- No

4.3.1.1 Please describe how you were infected (do not report any traceable information)

4.3.2 Did a doctor tell you that you (probably) have Corona/COVID-19 infection?

- Yes
- No

4.3.3 Are/ Were you in quarantine?

- No
- Yes, at home
- Yes, in the hospital
- Other, …

4.3.3.1 Describe your other situation with regard to quarantine, please

4.3.4 What medication (prescribed or not) did you take because of Corona?

- None
- Paracetamol
- Antibiotics
- Other, …

4.3.4.1 What other medication?

4.3.2.1 Did you get tested for Corona?

- Yes
- No
- I don't know

4.3.2.1.1 When did you test for Corona? (please estimate the date if you don't know precisely)

4.3.2.1.2 How was the test carried out?

- With a q-tip in the nose
- With a q-tip in the mouth
- Via blood test
- Other, …

4.3.2.1.3 What was the test result?

- Positive (Corona)
- Negative (no Corona)
- Haven't received a result yet
- I don't know

4.3.2.2 Were you hospitalized?

- Yes
- No

4.3.2.2.1 When were you hospitalized? (please provide an estimation if you don't know precisely)

4.3.2.2.2 Are/were you admitted to the ICU?

- Yes
- No

4.3.2.2.3 Was your breathing aided?

- Yes, with oxygen
- Yes, with a ventilation machine
- No
- Other, …

4.3.2.2.3.1 Please describe your other situation with regard to aided breathing

4.3.2.2.4 Were you discharged?

- Yes
- No

4.3.2.2.4.1 What is the discharge date? (please estimate the date if you don't know precisely)

4.3.5 Did you recover from the infection and are you in the same condition you were before Corona?

- Yes, fully recovered
- No, still recovering
- No, still ill
- Other, …

4.3.5.1 Please describe your situation with regard to your recovery (do not use any traceable information)

4.3.6 How many days have you been ill, since the first symptoms?

4.4 What score describes your situation JUST BEFORE the Corona outbreak the best?

This is a standard score which isn't made specially for FSHD patients. This could mean that the descriptions doesn't match your situation exactly. Please pick the option which fits your situation the best.

- No symptoms
- No significant disability, despite some symptoms; able to carry out all usual duties and activities
- Slight disability; unable to carry out all every-day activities, but able to look after own affairs without assistance
- Moderate disability; requiring some help, but able to walk without assistance
- Moderately severe disability; unable to walk without assistance and unable to attend to own bodily needs without assistance
- Severe disability; bedridden, incontinent and requiring constant nursing care and attention

4.5 What score describes your situation AT THE MOMENT the best?

- No symptoms
- No significant disability, despite some symptoms; able to carry out all usual duties and activities
- Slight disability; unable to carry out all every-day activities, but able to look after own affairs without assistance
- Moderate disability; requiring some help, but able to walk without assistance
- Moderately severe disability; unable to walk without assistance and unable to attend to own bodily needs without assistance
- Severe disability; bedridden, incontinent and requiring constant nursing care and attention

This is a standard score which isn't made specially for FSHD patients. This could mean that the descriptions doesn't match your situation exactly. Please pick the option which fits your situation the best.

4.6 Do you think Corona effects/ effected your FSHD symptoms?

- Yes
- No
- I don't know

4.7 Explain your answer, please.

4.8 Do you want to share other experiences with regards to Corona?

- Yes
- No

4.8.1 Note the experience you want to share, please.

Corona - Housemate 1-6

Down below are questions about your housemates (a maximum of six). You have more than six housemates. Please answer the questions for the six housemates you are the closest in contact with.

5.1 What age is your housemate?

5.2 What is your housemates gender?

- Male

- Female
- Other, …

5.3 Can you ask your housemate how tall he/she is?

- Yes
- No

5.3.1 Length

5.4 Can you ask your housemate what their weight is?

- Yes
- No

5.4.1 Weight

5.5 What is your relationship to your housemate?

- My partner
- My parent
- My kid
- My sister/brother
- Other family member
- Other, …

5.5.1 What is your other relationship to your housemate?

5.6 How often is your housemate at home?

- Whole week
- In the weekends, during the week lives my housemate somewhere else
- During the week, in the weekends live my housemate somewhere else
- Other, …

5.6.1 Please describe what part of the week your housemate is at home.

5.7 Is your housemate part of one or more risk groups?

- 70 years old or older
- Adult with respiratory illness under the treatment of a doctor
- Adult with chronical heart disease under treatment of a cardiologist
- Adult with poorly managed diabetes or diabetes with complications
- Adult on dialysis or with a donor kidney
- Adult with diminished immune response against infections caused by medication in connection with auto-immune disease
- Adult with organ or stem cell transplantation
- Adult with diminished immune response due to blood diseases
- Adult with diminished immune response due to spleen removal or disfunction of the spleen
- Adult with severe immune disfunction, which are treated
- Adult with cancer, which is treated in the past 3 months with radiation or chemotherapy
- Adult with HIV infection
- Adult with severe obesity
- Other,…

- No, my housemate doesn't fall into a risk group

5.7.1 Please describe in what other risk group your housemate part of.

5.8 Does your housemate have FSHD?

- Yes
- No
- I don't know

5.8.1 Does your housemate fill in this questionnaire himself/herself (as member of the registry)?

- Yes
- No

5.9 Did your housemate experience one of these symptoms down below, since the start of the Corona outbreak in The Netherlands mid-February?

- Dry cough without mucus
- Cough with mucus
- Taste loss
- Smell loss
- Flu-like feeling
- Dizziness
- Extra exhaustion (more than normal)
- Feverishness
- Fever (38℃ or higher)
- Headache
- No appetite
- Nausea
- Muscle pain
- Short of breath
- Breathing tightness
- Squeaking breathing
- Pain in the lungs/ with breathing
- Sore throat
- Stuffed nose
- Vomiting
- Ear pain
- Eye infection
- Other, …
- No symptoms

5.9.1 What other symptoms did your housemate experience?

5.9.2 When did your housemate first experience any of these symptoms? Please estimate the date if you don't know precisely.

5.9.3 How bad were the symptoms of your housemates?

- Minimal symptoms
- Few symptoms, having a severe cold but not sick
- Sick

- Severely sick

5.10 Was your housemate in contact with someone with Corona?

- Yes
- No

5.9.4 Do you think your housemate has/had Corona?

- Yes
- No
- Maybe

5.11.1 Where does your housemate expect this contamination came from?

- Yes
- No

5.11.1.1 Please describe how he/she got infected. (do not use any retraceable information)

5.11.2 Did a doctor tell your housemate that he/she (probably) has Corona/COVID-19 infection?

- Yes
- No

5.11.3 Is/ Was your housemate in quarantine?

- No
- Yes, at home
- Yes, in the hospital
- Other, …

5.11.3.1 Describe your housemates other situation with regard to quarantine, please

5.11.4 What medicine (prescribed or not) did your housemate take because of Corona?

- None
- Paracetamol
- Antibiotics
- Other, …

5.11.4.1 What other medicine?

5.11.2.1 Did your housemate get tested for Corona?

- Yes
- No
- I don’t know

5.11.2.1.1 When did your housemate get tested for Corona? (please estimate the date if you don’t know precisely)

5.11.2.1.2 How was the test carried out?

- With a q-tip in the nose
- With a q-tip in the mouth
- Via blood test

- Other, …

5.11.2.1.2.1 Describe how the test was carries out, please

5.11.2.1.3 What was the test result?

- Positive (Corona)
- Negative (no Corona)
- Haven’t received a result yet
- I don’t know

5.11.2.2 Was your housemate hospitalized?

- Yes
- No

5.11.2.2.1 When did your housemate get hospitalized? (please estimate if you don’t know precisely)

5.11.2.2.2 Is/was your housemate hospitalized at the IC?

- Yes
- No

5.11.2.2.3 Does/did your housemate get artificial respiration?

- Yes, oxygen
- Yes, respiration
- No
- Other, …

5.11.2.2.3.1 Describe the other situation with regard to artificial respiration, please

5.11.2.2.4 Did your housemate leave the hospital in the meantime?

- Yes
- No

5.11.2.2.4.1 When did your housemate leave the hospital? (please estimate the date if you don’t know precisely)

5.11.5 Did you recover from the infection and are you in the same condition you were before Corona?

- Yes, fully recovered
- No, still recovering
- No, still sick
- Other, …

5.11.5.1 Describe the situation with regard to his/her recovery, please. (don’t use any retraceable information)

5.11.6 How many days has your housemate been sick, since the first symptoms?

5.12 What score describes your housemates situation JUST BEFORE the Corona outbreak the best?

This is a standard score which isn’t made specially for FSHD patients. This could mean that the descriptions doesn’t match your situation exactly. Please pick the option which fits your situation the best.

- No symptoms
- No significant disability, despite some symptoms; able to carry out all usual duties and activities
- Slight disability; unable to carry out all every-day activities, but able to look after own affairs without assistance
- Moderate disability; requiring some help, but able to walk without assistance
- Moderately-severe disability; unable to walk without assistance and unable to attend to own bodily needs without assistance
- Severe disability; bedridden, incontinent and requiring constant nursing care and attention

5.13 What score describes your housemates situation AT THE MOMENT the best?

- No symptoms
- No significant disability, despite some symptoms; able to carry out all usual duties and activities
- Slight disability; unable to carry out all every-day activities, but able to look after own affairs without assistance
- Moderate disability; requiring some help, but able to walk without assistance
- Moderately-severe disability; unable to walk without assistance and unable to attend to own bodily needs without assistance
- Severe disability; bedridden, incontinent and requiring constant nursing care and attention

This is a standard score which isn’t made specially for FSHD patients. This could mean that the descriptions doesn’t match your situation exactly. Please pick the option which fits your situation the best.

5.8.2 Do you think Corona effects/ effected your housemates FSHD symptoms?

- Yes
- No
- I don’t know

5.8.2.1 Explain your answer, please.

5.14 Does your housemate want to share other experiences with regards to Corona?

- Yes
- No

5.14.1 Note the experience your housemate wants to share, please.

Concluding questions and complement

11.1 Do you want to share any important information about the corona infection or something else?

11.2 What question would you want to ask other participants of the registration?

11.3 Do you have any comments about the questionnaire?

Be careful, it is important that there are no names or other retractable information listed in the questionnaire. Is this the case, please change that before sending the questionnaire. Many thanks.

Dear participants, thank you for your time and participation for this research. We appreciate it a lot.

We would like to point out our second questionnaire, which is send to you about other illnesses besides your FSHD and vaccinations. Your answers to that questionnaire are necessary to translate your answers about Corona into results. We hope that you would fill in that questionnaire as well.

On behalf of the FSHD registration team, Wiecke van de Put, Hanneke Deenen, Anna Greco and Baziel van Engelen.
