# Supplementary material for: Living with facioscapulohumeral muscular dystrophy during the first two COVID-19 outbreaks: a repeated patient survey in the Netherlands
Source: Acta Neurol Belg. 2024 Jan 13;124(2):559–66. doi: 10.1007/s13760-023-02443-3 (PMC10965598; doi:10.1007/s13760-023-02443-3)
Supplement: Supplementary file 2 — Supplementary file2 (PDF 150 kb) [file 13760_2023_2443_MOESM2_ESM.pdf]

Appendix 2

Article Title: Living with facioscapulohumeral muscular dystrophy during the first two COVID-19 outbreaks, a repeated patient survey in the Netherlands

Journal Name: Acta Neurologica Belgica

Author names: Johanna C.W. Deenen[1,a,b], Joost Kools[1,a], Anna Greco[2,a,d], Renée Thewissen[2,a], Wiecke van de Put[a], Anke Lanser[c], Leo A.B. Joosten[d,e], Andre L.M.Verbeek[b], Baziel G. M. van Engelen[a], Nicol C. Voermans[a].

[1]Authors contributed equally; [2] Authors contributed equally;

[a]Department of Neurology, Donders Institute for Brain, Cognition and Behaviour, Radboud university medical center, PO Box 9101, 6500 HB Nijmegen, the Netherlands;

[b]Department for Health Evidence, Radboud university medical center, PO Box 9101, 6500 HB Nijmegen, the Netherlands;

[c]Patient Representative & Chairman FSHD Advocacy Group, Patient Organization for Muscular Disease Spierziekten Nederland, Lt. Gen. van Heutszlaan 6, 3743 JN Baarn, the Netherlands;

[d]Department of Internal Medicine, Radboud university medical center, PO Box 9101, 6500 HB Nijmegen, the Netherlands;

[e]Department of Medical Genetics, Iuliu Hatieganu University of Medicine and Pharmacy, Strada Victor Babeș 8, Cluj-Napoca 400347, Romania.

Corresponding author: Nicol.Voermans@radboudumc.nl

Correspondence author:

Nicol C. Voermans, Department of Neurology, Donders Institute for Brain, Cognition and Behaviour, Radboud university medical center, Radboud university medical center, PO Box 9101, 6500 HB Nijmegen, the Netherlands. Tel: 00 31 24 36 14 308, Fax: 00 31 24 36 35 135. Email: nicol.voermans@radboudumc.nl

Questions added to Survey 2 and 3.

- Did you complete a previous Corona questionnaire?
    - I completed a previous questionnaire
    - I am answering this questionnaire for the first time

- Who fills in this questionnaire?
    - Me
    - Spouse or family
    - Other,…

- Who is this other?

- What is your length in cm?

- What is your weight in kg?

- Is this domestic care or (also) personal care?
    - Domestic care
    - Also personal care (possibly combined with domestic care)

- Was it a voluntary isolation?
    - Voluntary isolation
    - Mandatory
    - Other

- Describe other isolation situation

- How much bodily exercise did you have during the Corona outbreak?
    - Substantially less bodily exercise than usual
    - Little less bodily exercise than usual
    - As much bodily exercise as usual
    - A bit more bodily exercise than usual
    - Substantially more bodily exercise than usual

- Who carried out the test?
    - Test street
    - General practitioner
    - Night/emergency center for general practice
    - Municipal medical assistance organisation
    - Regional medical assistance organisation
    - Hospital
    - Selftest
    - Commercial party
    - Do not know

Questions added to Survey 3.

- Did you get tested for Corona since the start of the pandemic (early 2020)?
  - Yes
  - No
  - I don’t know

- How often were you tested since the start of the pandemic (early 2020)?

- How often was the test positive since the start of the pandemic (early 2020)?

- Was this a re-contamination?
  - Yes
  - No
  - I don’t know

- When were you tested the first time and the test result was positive since the start of the pandemic (early 2020)?

- When were you tested the second time and the test result was positive since the start of the pandemic (early 2020)??

- When did you last tested positive since the start of the pandemic (early 2020)?

<u>Questions regarding housemates</u>

- How often was your housemate tested since the start of the pandemic (early 2020)?

- How often was the test positive since the start of the pandemic (early 2020)?

- Was this a re-contamination?
  - Yes
  - No
  - I don’t know

- When did your housemate get tested and the test result was positive for the first time since the start of the pandemic (early 2020)?

- When did your housemate get tested the second time and the test result was positive since the start of the pandemic (early 2020)?

- When did your housemate get the last positive test since the start of the pandemic (early 2020)?
