# Supplementary material for: Living with facioscapulohumeral muscular dystrophy during the first two COVID-19 outbreaks: a repeated patient survey in the Netherlands
Source: Acta Neurol Belg. 2024 Jan 13;124(2):559–66. doi: 10.1007/s13760-023-02443-3 (PMC10965598; doi:10.1007/s13760-023-02443-3)
Supplement: Supplementary file 3 — Supplementary file3 (PDF 143 kb) [file 13760_2023_2443_MOESM3_ESM.pdf]

Appendix 2.

Article Title: Living with facioscapulohumeral muscular dystrophy during the first two COVID-19 outbreaks, a repeated patient survey in the Netherlands

Journal Name: Acta Neurologica Belgica

Author names: Johanna C.W. Deenen[1,a,b], Joost Kools[1,a], Anna Greco[2,a,d], Renée Thewissen[2,a], Wiecke van de Put[a], Anke Lanser[c], Leo A.B. Joosten[d,e], Andre L.M.Verbeek[b], Baziel G. M. van Engelen[a], Nicol C. Voermans[a].

[1]Authors contributed equally; [2] Authors contributed equally;

[a]Department of Neurology, Donders Institute for Brain, Cognition and Behaviour, Radboud university medical center, PO Box 9101, 6500 HB Nijmegen, the Netherlands;

[b]Department for Health Evidence, Radboud university medical center, PO Box 9101, 6500 HB Nijmegen, the Netherlands;

[c]Patient Representative & Chairman FSHD Advocacy Group, Patient Organization for Muscular Disease Spierziekten Nederland, Lt. Gen. van Heutszlaan 6, 3743 JN Baarn, the Netherlands; [d]Department of Internal Medicine, Radboud university medical center, PO Box 9101, 6500 HB Nijmegen, the Netherlands; [e]Department of Medical Genetics, Iuliu Hatieganu University of Medicine and Pharmacy, Strada Victor Babeș 8, Cluj-Napoca 400347, Romania.

Corresponding author: Nicol.Voermans@radboudumc.nl

Correspondence author:

Nicol C. Voermans, Department of Neurology, Donders Institute for Brain, Cognition and Behaviour, Radboud university medical center, Radboud university medical center, PO Box 9101, 6500 HB Nijmegen, the Netherlands. Tel: 00 31 24 36 14 308, Fax: 00 31 24 36 35 135. Email: nicol.voermans@radboudumc.nl

Appendix 2 Frequency and burden of COVID-19 related stressors

Stressor	Frequency, N(%)			Median burden [IQR]			Mean burden (SD)		
	S1	S2	S3	S1	S2	S3	S1	S2	S3
Having COVID-19 symptoms or symptoms that could be related	48 (22.9)	40 (21.5)	52 (25.4)	3 [2-4]	3 [2-3]	3 [1-3]	2.87 (1.12)	2.55 (1.08)	2.61 (1.25)
Having COVID-19 symptoms or symptoms that could be related, in family members, friends, loved ones or colleagues	72 (34.3)	62 (33.3)	101 (49.3)	3 [2-3]	3 [2-3]	3 [2-3]	2.79 (1.15)	2.55 (1.07)	2.63 (1.12)
Being at risk for an infection (e.g. at work, in the supermarket)	117 (55.7)	108 (58.1)	127 (62.0)	3 [2-3]	3 [2-3]	3 [1-3]	2.77 (1.00)	2.54 (1.09)	2.47 (1.10)
Being at risk for a serious course of the disease in case of a COVID-19 infection	97 (46.2)	78 (41.9)	109 (53.2)	3 [2-4]	3 [2-4]	3 [2-4]	3.17 (1.19)	2.85 (1.30)	2.92 (1.29)
Family, friends or loved ones being at risk for a serious course of the disease in case of a COVID-19 infection	133 (63.3)	89 (47.8)	116 (56.6)	3 [3-4]	3 [2-4]	3 [2-4]	3.21 (1.18)	2.93 (1.19)	2.90 (1.32)
Problems with access to healthcare, medication or sanitation	112 (53.3)	100 (53.8)	110 (53.7)	3 [1-3]	3 [2-3]	3 [1-3]	2.56 (1.24)	2.49 (1.09)	2.46 (1.22)
Feeling restricted to leave your home	156 (74.3)	113 (60.8)	138 (67.3)	3 [1-4]	2 [1-3]	3 [1-3]	2.56 (1.28)	2.39 (1.08)	2.54 (1.21)
Loss of social contact and social events [a]	192 (91.4)	160 (86.0)	183 (89.3)	3 [2-4]	3 [2-4]	3 [3-4]	2.89 (1.20)	2.81 (1.09)	3.17 (1.06)
Family, friends or loved ones are at the hospital and you are restricted in visiting them [b]	129 (61.4)	105 (56.5)	129 (62.9)	3 [3-4]	3 [3-4]	3 [2-4]	3.23 (1.16)	3.03 (1.00)	3.19 (1.21)
Unable to attend a funeral of a loved one [b]	59 (28.1)	62 (33.3)	66 (32.2)	4 [3-4]	3 [2-4]	4 [2-4]	3.57 (1.16)	3.06 (1.25)	3.27 (1.26)
Family, friends or loved ones working in vital professions	103 (49.0)	103 (55.4)	108 (52.7)	2 [1-3]	2 [1-3]	2 [1-3]	2.43 (1.13)	2.20 (1.05)	2.31 (1.00)
Less physical activity than usual	141 (67.1)	117 (62.9)	144 (70.2)	3 [2-4]	3 [2-3]	3 [2-4]	2.87 (1.13)	2.74 (1.08)	2.81 (1.14)
Difficulties combining work with childcare	17 (8.1)	12 (6.5)	14 (6.8)	2 [1-3]	3 [1-4]	3 [1-3]	2.06 (1.09)	2.75 (1.29)	2.57 (1.22)
Tensions at home or family conflict	68 (32.4)	57 (30.6)	55 (26.8)	2 [1-3]	2 [1-3]	2 [1-3]	2.10 (1.07)	2.26 (1.17)	2.36 (1.16)
Increased workload or work-related obstacles	55 (26.2)	38 (20.4)	53 (25.9)	2 [1-3]	3 [2-4]	2 [1-3]	2.27 (1.22)	2.81 (1.27)	2.28 (1.04)
(Threat of) job loss, insolvency of a private company, for yourself or someone in your household	20 (9.5)	18 (9.7)	13 (6.3)	3 [1-4]	3 [2-3]	4 [2.5-4]	2.70 (1.45)	2.44 (0.98)	3.38 (1.33)
Problems obtaining basic needs and services	81 (38.6)	57 (30.6)	66 (32.2)	2 [1-3]	2 [1-3]	2 [1-3]	2.35 (1.11)	2.11 (1.03)	2.23 (1.05)
COVID-19 related media coverage [a]	189 (90.0)	168 (90.3)	183 (89.3)	2 [1-3]	2 [1-3]	2 [1-3]	2.34 (1.14)	2.21 (1.19)	2.38 (1.16)

A: Stressors which had the highest frequency. B: Stressors which had the highest burden. S1 = Survey 1. S2= Survey 2. S3= Survey 3.
